# Longitudinal progression, metrics, age-dependence, and modifiers of ataxia severity in SCA27B: a multicentre study of 219 patients

**DOI:** 10.1016/j.ebiom.2026.106437

**Published:** 2026-08-15

**Authors:** Andreas Traschütz, Ralf-Dieter Hilgers, Friedrich Erdlenbruch, Christel Depienne, Thomas Wirth, Clarisse Delvallée, Astrid Nümann, Catherine Ashton, David Pellerin, Elisabetta Indelicato, Felix Heindl, Mathilde Renaud, Max Borsche, Marcus Grobe-Einsler, Jennifer Faber, Thomas Klockgether, Ludger Schöls, Bernard Brais, Mathieu Anheim, Dagmar Timmann, Matthis Synofzik

**Affiliations:** a Division Translational Genomics of Neurodegenerative Diseases, Hertie Institute for Clinical Brain Research and Center of Neurology, University of Tübingen, Tübingen, Germany; b German Center for Neurodegenerative Diseases (DZNE), University of Tübingen, Tübingen, Germany; c Department of Neurology and Epileptology, Hertie-Institute for Clinical Brain Research and Center of Neurology, University of Tübingen, Tübingen, Germany; d RWTH Aachen University, Institute of Medical Statistics, Aachen, Germany; e Medical Faculty, Sigmund Freud Private University, Vienna, Austria; f Department of Neurology and Center for Translational Neuro- and Behavioral Sciences (C-TNBS), Essen University Hospital, University of Duisburg-Essen, Essen, Germany; g Institute of Human Genetics, University Hospital Essen, University of Duisburg-Essen, Essen, Germany; h Service de Neurologie, Hôpitaux Universitaires de Strasbourg, Strasbourg, France; i Département de Médecine Translationnelle et Neurogénétique, Institut de Génétique et de Biologie Moléculaire et Cellulaire, INSERM-U964/CNRS-UMR7104/Université de Strasbourg, Illkirch-Graffenstaden, France; j Institut des Neurosciences de Strasbourg, Université de Strasbourg, Strasbourg, France; k Charité - Universitätsmedizin Berlin, Corporate Member of Freie Universität Berlin and Humboldt-Universität zu Berlin, Department of Neurology, Berlin, Germany; l Experimental and Clinical Research Center, A Cooperation Between Max Delbrück Center for Molecular Medicine in the Helmholtz Association and Charité - Universitätsmedizin Berlin, Berlin, Germany; m Department of Neurology, Royal Perth Hospital, Perth, Western Australia, Australia; n Department of Neurology and Neurosurgery, Montreal Neurological Hospital and Institute, McGill University, Montreal, QC, Canada; o Department of Neurology, Medical University of Innsbruck, Innsbruck, Austria; p Department of Neurology, University Hospital, Ludwig-Maximilians University, Munich, Germany; q INSERM-U1256 NGERE, Université de Lorraine, Nancy, France; r Service de Neurologie, CHRU de Nancy, Nancy, France; s Service de Génétique Clinique, CHRU de Nancy, Nancy, France; t Institute of Neurogenetics, University of Lübeck and University Hospital Schleswig-Holstein, Lübeck, Germany; u Department of Neurology, University of Lübeck and University Hospital Schleswig-Holstein, Campus Lübeck, Lübeck, Germany; v Department of Neurology, University Hospital Bonn, Bonn, Germany; w German Center for Neurodegenerative Diseases (DZNE), Bonn, Germany; x Department of Neurology, Center for Movement Disorders and Neuromodulation, Heinrich Heine University Düsseldorf, Düsseldorf, Germany; y Department of Parkinson's Disease, Sleep and Movement Disorders, Center for Neurology, University Hospital Bonn, Bonn, Germany; z Department of Neuroradiology, University Hospital Bonn, Bonn, Germany; aa Department of Neurodegeneration, Hertie Institute for Clinical Brain Research and Center of Neurology, University of Tübingen, Germany

**Keywords:** Ataxia, SCA27B, Natural history, Progression, Modifiers, Trials

## Abstract

**Background:**

Spinocerebellar Ataxia 27B (SCA27B) is a novel, frequent and likely treatable late-onset autosomal-dominant ataxia caused by GAA repeat-expansions in *FGF14*. For understanding disease evolution and imminent trial planning, metrics of the most widely used clinical outcome assessment (Scale for the Assessment and Rating of Ataxia/SARA), longitudinal progression and modifiers thereof are warranted.

**Methods:**

Multicentre intercontinental observational study (2015–2024) of 661 assessments from 219 patients with SCA27B (age: 68 ± 10 years; SARA: 9 ± 6 points) with item-level distribution-based analyses to characterise SARA metrics relative to ageing-related impairment in 390 healthy controls; and linear mixed-effects modelling to determine longitudinal progression and demographic or genetic modifiers.

**Findings:**

Ataxia severity in SCA27B as assessed by SARA was primarily attributable to gait, stance, and lower-limb impairment; other ataxia domains scored ≤1 SARA point in 79–94% of patients. Discrimination of SCA27B motor performance from controls decreased with age due to ageing-related motor variability captured by SARA, thus limiting potential metric response windows for symptomatic treatments. Disease progression was faster in the presence of interfering ageing-related comorbidities in 14 (6%) patients. Overall longitudinal progression of SCA27B was 0.54 SARA points/year [95% CI: 0.37–0.71]. Expansions of (GAA)_> 180_ repeats were frequent also on the shorter allele (n = 18 (8%), range: 196–348 repeats), and associated with faster progression (+1.6 SARA points/year, [95% CI: 0.9–2.2]), including also otherwise less affected ataxia domains speech and sitting.

**Interpretation:**

Disease progression in SCA27B is characterised by mild progression, ageing-related motor variabilities and comorbidities, and associated with repeat size on both alleles.

**Funding:**

Else-Kröner-Fresenius-Stiftung, EU, DFG, BMBF, CIHR, NAF, Ataxia-UK, CSC.

**Research in context**

**Evidence before this study**

Spinocerebellar Ataxia 27B (SCA27B) is a recently identified, frequent and likely treatable cause of late-onset ataxia due to GAA repeat expansions in one of two alleles of the *FGF14* gene. Small, mostly cross-sectional studies in the PubMed database until August 2025 have described the clinical phenotype and provided preliminary estimates of disease progression in SCA27B. However, imminent trial planning in SCA27B requires a better understanding of longitudinal disease progression; of outcome metrics to best measure progression, in particular with the Scale for the Assessment and Rating of Ataxia (SARA) as most widely used clinical outcome assessment; and of potential disease modifiers to better predict progression. Further, it remains unclear how ageing-related motor impairment and comorbidities affect the assessment of disease progression and outcome metrics in the elderly population suffering from SCA27B.

**Added value of this study**

This large multicentre study including 219 patients with SCA27B provides a robust estimate of longitudinal progression (0.54 SARA points per year), and identifies impairment of gait and stance as strongest contributors to disease severity and progression. The study further demonstrates that frequent ageing-related comorbidity interferes with the assessment of disease severity in populations with SCA27B, and that capture of ageing-related motor variability impairs SARA's discrimination of motor performance between patients with SCA27B and healthy controls, thus limiting its metric response window for future symptomatic trials. Importantly, the study identifies repeat length on the shorter *FGF14* allele as a potential genetic modifier of progression, suggesting that repeat size of the shorter allele is associated with a faster disease course, even already above only >180 GAA repeats. Together, these findings define clinical, ageing-related, and genetic factors that are crucial for the design and interpretation of upcoming interventional studies in SCA27B.

**Implications of all the available evidence**

This study highlights that disease progression in SCA27B, while overall mild, is primarily attributable to impairment in gait and balance, and influenced by ageing-related motor variability and comorbidity. Future clinical trials should thus prioritise outcome measures with high sensitivity in the gait and balance domains, such as digital gait metrics or balance-focused scales, and apply careful patient selection and stratification. Repeat expansions on the shorter *FGF14* allele as a modifier of progression suggest a bi-allelic contribution to disease mechanisms that warrants further investigation in larger genetic and clinical studies.

## Introduction

Intronic GAA repeat expansions in *FGF14* have been recently identified as genetic cause of Spinocerebellar Ataxia 27B (SCA27B),^1^^,^^2^ by now already widely acknowledged as the most frequent cause of late-onset autosomal dominant cerebellar ataxia in many populations.^3^, ^4^, ^5^, ^6^, ^7^ Moreover, SCA27B may be amenable to already available drug treatment, with accumulating observational evidence indicating a treatment effect of 4-aminopyrdine (4-AP) in SCA27B.^1^^,^^8^, ^9^, ^10^, ^11^ Taken together, the high prevalence plus probable treatability of SCA27B underline the urgent need for a timely understanding of optimal outcome metrics, longitudinal progression, and potential modifiers thereof–not only to better understand this disease, but also to guide imminent trial planning.

The Scale for the Assessment and Rating of Ataxia (SARA) presents the most common clinical outcome assessment (COA) in genetic ataxias,^12^, ^13^, ^14^ but available data on its metrics or progression in SCA27B is limited to cross-sectional data and/or small cohorts.^4^^,^^8^^,^^15^ Moreover, existing data suggests that SCA27B presents a rather mild ataxia that could be difficult to capture by the SARA in clinical trials.^4^^,^^7^^,^^8^ Implementation of the SARA as COA could be further complicated by the predominant manifestation of SCA27B in late-adult and elderly patients, in whom ageing-related motor impairment or comorbidities become more common.^8^^,^^16^^,^^17^ Such SCA27B-unrelated factors may interfere with the clinical assessment of ataxia severity per se, and/or limit the window of responsiveness of the SARA to treatment effects (given that a SARA score increased due to general ageing or other comorbidities might limit the change in SARA score achievable by any treatment in SCA27B).

Compiling a large, intercontinental, multicentre, cross-sectional and longitudinal cohort of 219 patients with SCA27B assessed with the SARA, we here (i) analyse metrics of the SARA by distribution-based analysis at the item-level; (ii) compare its responsiveness to a large cohort of age-matched healthy controls up to the 8th decade of age; (iii) estimate longitudinal disease progression in SCA27B after controlling for comorbidity that may confound SARA-based ataxia severity and progression estimates; and (iv) identify modifiers of disease progression, including the size of the GAA repeats on the shorter allele. These results will be important to better understand this recently identified disease; and in particular to inform data-driven design of imminently upcoming SCA27B trials.

## Methods

### Study cohort

Patients with SCA27B—prospectively or retrospectively genetically confirmed (≥250 uninterrupted GAA repeats) since the discovery of the genetic cause of the disease^1^—were collected from nine centres in Europe and North America (n = 219; for details across sites, see Table S1). To characterise disease evolution, all consecutive patients with SCA27B until January 2024 with at least one historical or prospective assessment with the SARA as key COA for ataxia,^12^ documented at the item-level, were included (observation period across centres: 2015–2024). The SARA yields total scores from 0 (no ataxia) to 40 (most severe ataxia), based on clinical examination across eight items (gait: 0–8; stance: 0–6 and sitting: 0–4; speech: 0–6; finger chase, nose-finger test, fast alternating hand movements, and heel-shin slide: 0–4 points). Patients and their medical records, respectively, were systematically reviewed by the local physician for all previous SARA assessments to avoid sampling bias; for the presence of non-ataxia features; and for further clinical information that might influence disease evolution of SCA27B (=demographics, age of onset, visit dates), or otherwise interfere with the assessment of motor impairment, e.g. diseases unrelated to SCA27B (=comorbidities), or effects due to treatment with 4-AP or acetazolamide. Sex was not self-reported for the purposes of this study. It was extracted by the local investigators from the administrative patient records of each participating centre, where it is registered as legal/administrative sex on the basis of health-insurance registration data; gender was not assessed. Data was collected and linked using a common structured data sheet, including prespecified queries about genetics, demographics, comorbidity, treatment, and item-level SARA scores.

### Genotyping

Genetic testing for GAA repeat expansions in *FGF14* was performed by long-read sequencing, or sequential testing by long-range PCR, repeat-primed PCR, and Sanger sequencing, as previously described.^1^^,^^18^^,^^19^ GAA repeat number was determined for both the longer and the shorter allele in all patients, using a pathogenic cutoff of ≥250 uninterrupted GAA repeats on the longer allele.^1^^,^^2^^,^^15^

### Ethics

This study adhered to the 2024 Declaration of Helsinki, data collection was approved by the institutional review board of the medical faculty of the University of Tübingen (598/2011BO1), and patients at all contributing sites provided written informed consent.

### Statistics

To determine how disease severity and its progression in SCA27B is reflected by the SARA across different ataxia domains, its metrics were characterised by distribution-based analysis of its total score as well as its item subscores across all baseline assessments. This allowed to analyse the relative share of patients at each item severity level and potential floor/ceiling effects. To characterise how assessment by the SARA is affected by general, i.e. SCA27B-independent, ageing-related motor impairments -as might be expected in late-adult and elderly populations like SCA27B-, the distribution of the SARA and its items in all 219 patients with SCA27B at baseline were next compared to cross-sectional assessments in 390 healthy elderly controls between 18 and 79 years of age (mean: 47.5 ± 17.1 years) from an independent validation cohort, also assessed by experienced clinical raters as part of distinct SARA validation studies and in non-mutation carriers in prospective natural history studies of presymptomatic spinocerebellar ataxias.^20^ This comparison was stratified by the decade of each subject's age at SARA assessment, and the area under the curve (AUC) was calculated as effect size for the discrimination between patients with SCA27B and healthy controls. We thereby aimed to quantify the potential “window of responsiveness” of the SARA in SCA27B, i.e. the delta between the SARA of the disease state (SCA27B) and the SARA of the general, ageing-related healthy state in the corresponding age decade.

Longitudinal disease progression in SARA for SCA27B was analysed by linear mixed-effect modelling (LMEM, restricted maximum likelihood, covariance structure: variance components, Kenward– Roger degrees of freedom) with random effects on intercept (score at baseline) and slope (points per year of follow-up).^21^ LMEM was performed both in the overall cohort, and in the clinically assumed most trial-relevant subcohort of ambulatory patients, defined by a SARA gait item ≤3, i.e. milder than levels requiring intermittent support or walking aids. To obtain valid progression estimates, clinical records of patients exceeding the 95th percentile of SARA total scores per decade of age, and/or the 95th percentile of population intercepts and slopes of a preliminary LMEM were manually reviewed for potential outliers before the final analysis. These patients were excluded from all final LMEM analyses if SCA27B-independent diseases, multi-morbidity with reduced functional status, and/or recent health events were identified that likely interfered with the SARA at the time of motor assessment, as evaluated independently by two assessors (A.T., M.Sy.). Two separate LMEMs were calculated for (i) the SARA total score and (ii) its item-level subscores to explore and compare disease evolution in respective ataxia effector domains. For the SARA total score, LMEM included ataxia severity (SARA) and disease duration at baseline as well as 4-AP treatment as covariates; and sex, age of onset, and GAA repeat number as exploratory independent factors, selected a priori based on expert knowledge and previous literature. For the item-level analyses, we applied a parsimonious modelling approach by restricting models to factors that had shown relevant associations at the total score level, in order to avoid overfitting and maintain interpretability. The item-level model included the normalised subscores in a joint LMEM for simultaneous assessment of most relevant item progression. The results were retransformed for presentation purposes.

Descriptive and test statistics, linear regression analysis, Spearman correlations, and distribution-based analyses, including receiver operating characteristics, were calculated using Prism 10 (GraphPad Software, La Jolla, CA). LMEM was performed with SAS, version 9.4 (procedure MIXED, NLMIXED). Data are shown as mean ± standard deviation, interquartile range (IQR), 95% confidence intervals (95% CI), or standard error of the mean (SEM). No formal sample-size calculation was performed for this observational cohort.

### Role of funders

The funders of this study had no role in study design, data collection, data analysis, data interpretation, the writing of the report, or the decision to submit the paper for publication. The corresponding author had full access to all the data in the study and had final responsibility for the decision to submit for publication.

## Results

### Cohort characteristics

**Fig. 1:**
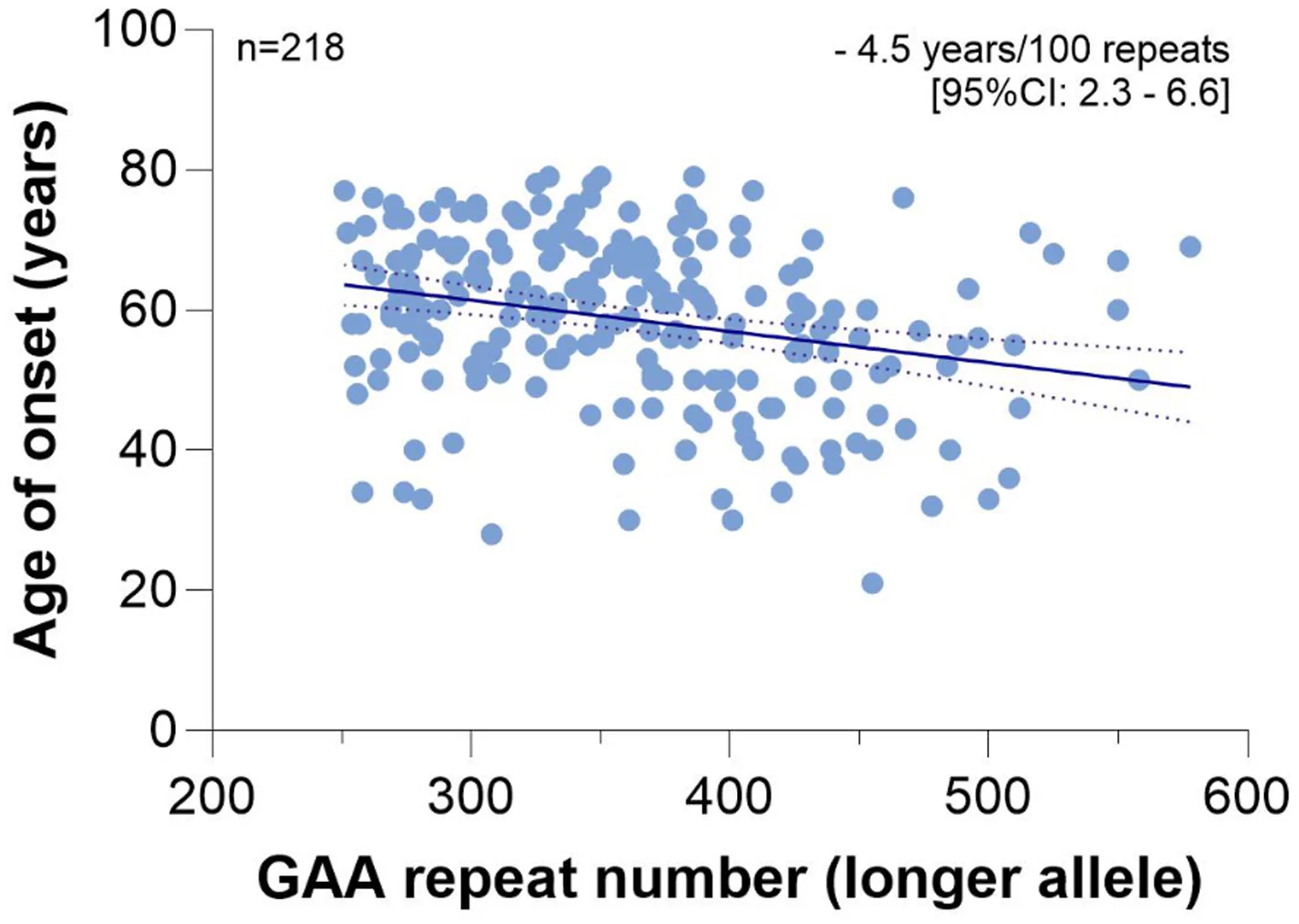
Association between age of onset and GAA repeat number on the longer allele in 218 patients with SCA27B, excluding one patient with an extreme value of 937 repeats. Solid line indicates linear regression, dashed lines reflect 95% confidence bands of the best-fit line.

This study included 661 SARA assessments from 219 patients (101 female) with genetically confirmed SCA27B across nine centres in Germany (Tübingen: n = 54; Essen: n = 34; Berlin: n = 21; Munich: n = 12; Lübeck: n = 7), Canada (Montreal: n = 44), France (Strasbourg: n = 26; Nancy: n = 11), and Austria (Innsbruck: n = 10), including 116 patients across all centres with longitudinal assessments over an average of 4 [IQR: 3–6] follow-up visits in 4.7 ± 3.4 years (range: 0.3–15.4; Table 1, see Table S1 for details across sites). The full study cohort spanned a wide range of patient age (68 ± 10 years, range: 24–86), age of onset (59 ± 12 years, range: 21–79), disease duration (9 ± 7 years, range: 0–32), and ataxia severity at baseline (SARA: 9 ± 6 points, range: 0–34), thus allowing for representative analyses of SARA metrics and progression in SCA27B. Mild to moderate impairment of the vibration sense at the ankle indicating sensory neuropathy (93/215 patients assessed, 43%) and an impaired head-impulse test indicating vestibular dysfunction (20/100, 20%) were frequent non-ataxia features known in SCA27B; less common non-ataxia features were mild cognitive impairment (19/216, 8%), signs of pyramidal tract involvement in the legs (hyperreflexia, spasticity or extensor plantar response: 16/216, 7%), mild postural or head tremor (12/216, 6%), and mild parkinsonism (11/219, 5%; see Table S1 for details). Genetically, the number of GAA repeats on the longer allele ranged from 251 to 937 repeats (median: 356, IQR: 303–407) and was negatively associated with the age of onset, decreasing the average age of onset by 4.5 years [95% CI: 2.3–6.6] per 100 repeats between 250 and 600 repeats (linear regression, *p* < 0.001; n = 218, i.e. excluding one outlier patient with 937 repeats, Fig. 1).

**Table 1:** Cohort characteristics at baseline visit.

	Total cohort (n = 219)
Age (years)	68 ± 10 (24–86)
Sex	101 female (46%)
	118 male (54%)
	0 other (0%)
Age of onset (years)	59 ± 12 (21–79)
Disease duration (years)	9 ± 7 (0–32)
Ataxia severity (SARA score)	9 ± 6 (0–34)
Number of FGF14 GAA repeats	
Longer allele	356 [303–407]
Shorter allele	16 [9–53]
Drug treatment	
On 4-AP or 3,4-DAP	40 (18%)
On Acetazolamide	14 (6%)
Longitudinal follow-up	
Number of patients	116 (53%)
Number of follow-up visits	4 [3–6]
Follow-up time (years)	4.7 ± 3.4 (0.3–15.4)
Non-ataxia features	
Impaired vibration sense at ankle	93/215 (43%)
Impaired head impulse test	20/100 (20%)
Pyramidal signs in the legs	16/216 (7%)
Paresis	3/218 (1%)
Postural or head tremor	12/216 (6%)
Parkinsonism	11/219 (5%)
Cognitive impairment	19/216 (8%)

Data presented as mean ± standard deviation (range), median [interquartile range], or number of patients (%cohort).

4-AP = 4-aminopyridine; 3,4-DAP = 3,4-diaminopyridine.

### Item-level SARA metrics

**Fig. 2:**
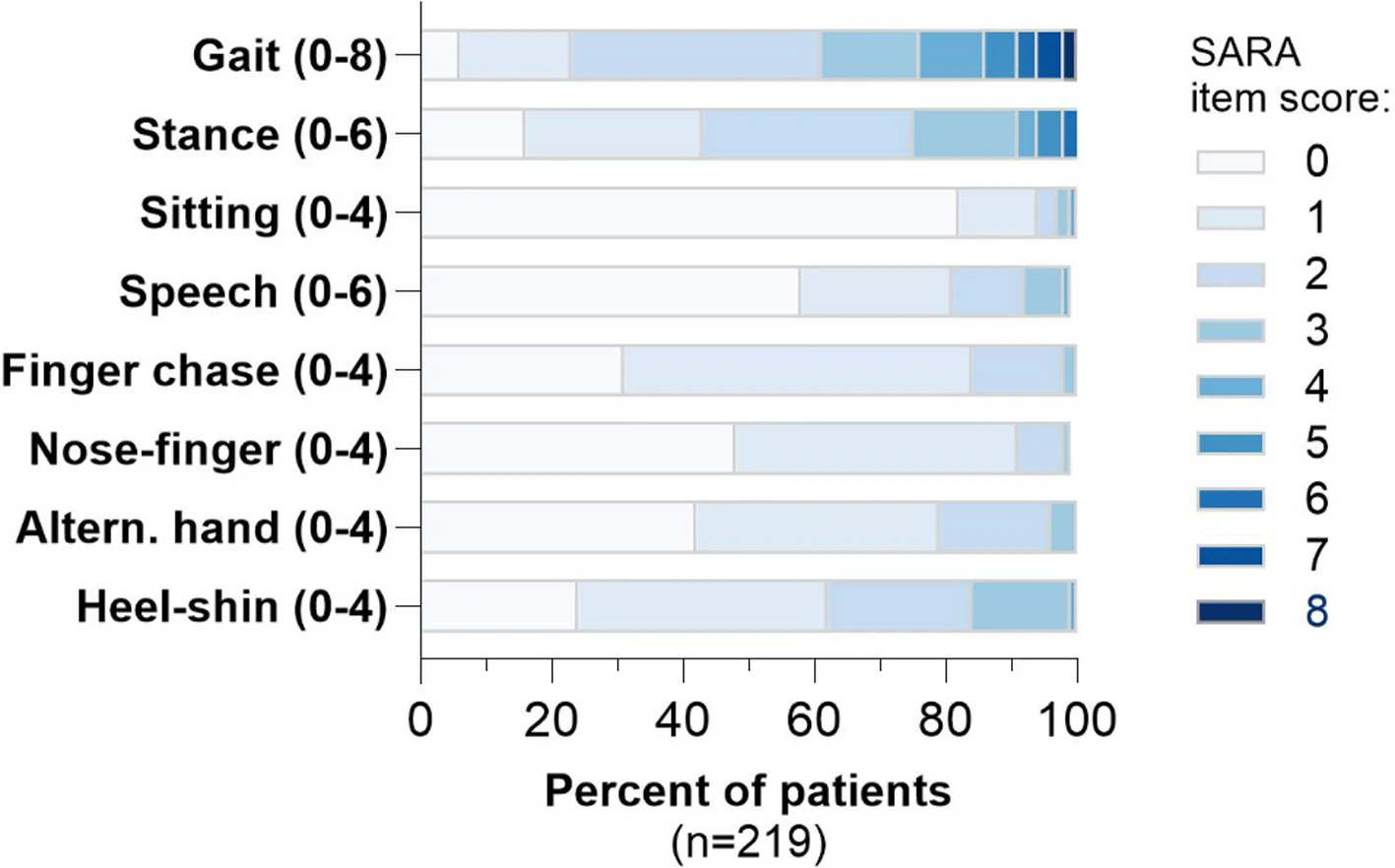
Disease severity in SCA27B across ataxias domains, as measured by the distribution of scores across SARA items among 219 patients. Numbers in brackets indicate maximum scoring range per item. No statistical test was applied.

SARA total score and all item subscores at baseline correlated with disease duration, thus capturing disease severity over time, albeit with only small-to-moderate effect sizes, indicating that factors other than just disease duration influence the SARA levels (Spearman correlation, SARA: rho = 0.30 [95% CI: 0.17–0.42], *p* < 0.001; items: rho = 0.17–0.32 [95% CI: 0.03–0.44], all *p* ≤ 0.030; Fig. S2). Distribution of severity degrees per item, i.e. score levels per item, showed that ataxia severity in SCA27B was mainly captured by SARA items *gait* and *stance* in the balance domain, and the *heel-shin* item in the lower limb domain (Fig. 2). In items *gait* and *stance*, at least 50% of patients exceeded a subscore of 2; and 24% and 10% had subscores of 4 or higher, thus requiring at least intermittent support for gait and stance, respectively. In contrast, all SARA items apart from gait showed considerable floor effects, and patients with SCA27B predominantly scored ≤1 in other axial (sitting: 94%, speech: 81%) and upper limb items (finger–chase: 84%, nose-finger: 91%; diadochokinesia: 79%).

### SARA scores in SCA27B relative to healthy controls across age groups

**Fig. 3:**
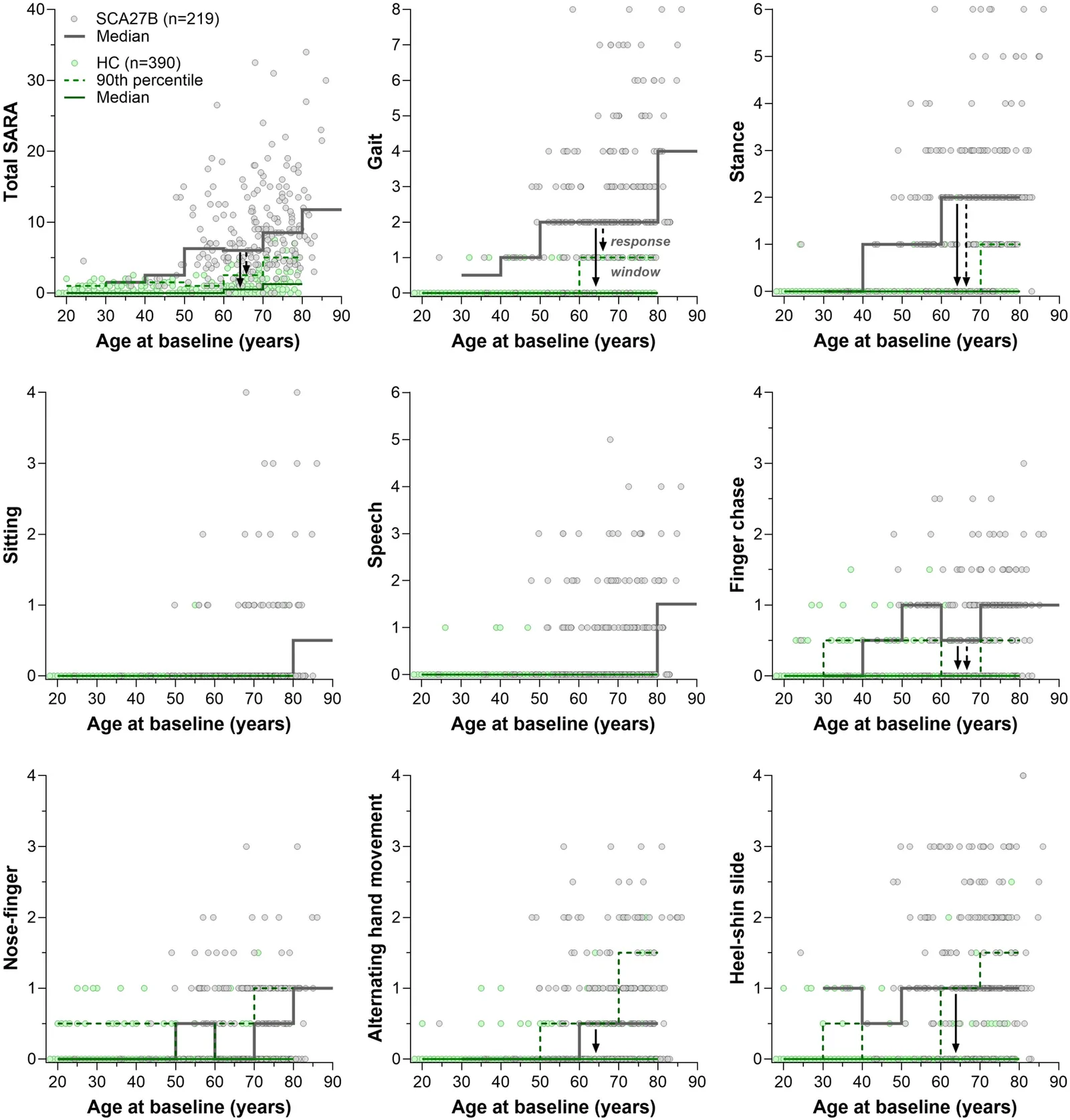
Comparison of SCA27B severity to healthy ageing-related motor impairment. SARA total score and item scores of 219 patients with SCA27B at the age of baseline assessment vs. normative reference values in 390 healthy aged controls. Solid lines and dashed lines reflect medians and 90th percentile calculated per decade of age, respectively. The effect size of the discrimination between patients with SCA27B and controls by the SARA worsened with age, with AUCs of 0.97 (50–60 years), 0.95 (60–70 years), and 0.91 (70–80 years), respectively, for the total SARA score (receiver operating characteristics, all *p* < 0.001). This difference between patients with SCA27B and age-stratified controls corresponds to the possible response window for a change in SARA in a hypothetical trial of a symptomatic treatment.

We next explored how motor impairments in general ageing, i.e. unrelated to SCA27B (or any other specific ataxia or other neurologic disease), might influence the SARA levels; and determined the relative delta in SARA levels in SCA27B vs. general ageing. For this, we compared the cross-sectional distribution of the SARA and its items in the SCA27B cohort to a large cohort of 390 healthy controls in the respective same age decade (Fig. 3). SARA total scores showed a consistent difference between patients with SCA27B and controls across decades, indicated by the fact that the progression of ataxia severity in SCA27B exceeded the average ageing-related increase of motor impairment (e.g. median [IQR] at 40–50 years: 2.5 [1.5–11] vs. 0 [0–1] points; 70–80 years: 8.5 [5–12.5] vs. 1.25 [0–3] points). However, the inter-individual variability of motor impairment—as assessed by the SARA—increased in healthy aged controls, leading to a decreasing difference between item levels of patients with SCA27B and controls with increasing age (e.g. median [IQR] of patients with SCA27B vs. 90th percentile in controls; 50–60 years: 6 [3.5–12] vs. 1 point; 60–70 years: 6 [4–10] vs. 2.5 points; 70–80 years: 8.5 [5–12.5] vs. 5 points). Accordingly, the effect size of the discrimination between patients with SCA27B and controls by the SARA worsened with age (receiver operating characteristics, AUC: 0.97 (50–60), 0.95 (60–70), and 0.91 (70–80), respectively, though all *p* < 0.001). This decreasing difference, which reflects the potential treatment-related improvement delta SARA in SCA27B relative to merely ageing-related SARA levels, suggests that the metric response window of the SARA decreases with higher age in patients with SCA27B.

At the item level, only SARA items *gait* and *stance* showed up to a 2-point difference between the median of patients with SCA27B vs. controls, while items *finger–chase* and *heel-shin* had a maximum 1-point difference (Fig. 3), indicating a potentially higher response window for gait and stance than for the latter appendicular items. Such larger differences for gait and stance between SCA27B and controls were observed across all age groups, thus suggesting even an age-independent, general aspect of SCA27B disease. This was further corroborated by the observation that ageing-related motor impairments increased in particular the variability of the appendicular items of the SARA. For example, a 90% percentile of up to 1.5 SARA points was observed in healthy controls for the items *diadochokinesia* and *heel-shin* above age 70 years. In contrast to gait and stance, this ageing-related variability in the appendicular items exceeded even the median item level observed in the patients with SCA27B.

### Progression modelling and interference by frequent comorbidity in late-adult populations

**Fig. 4:**
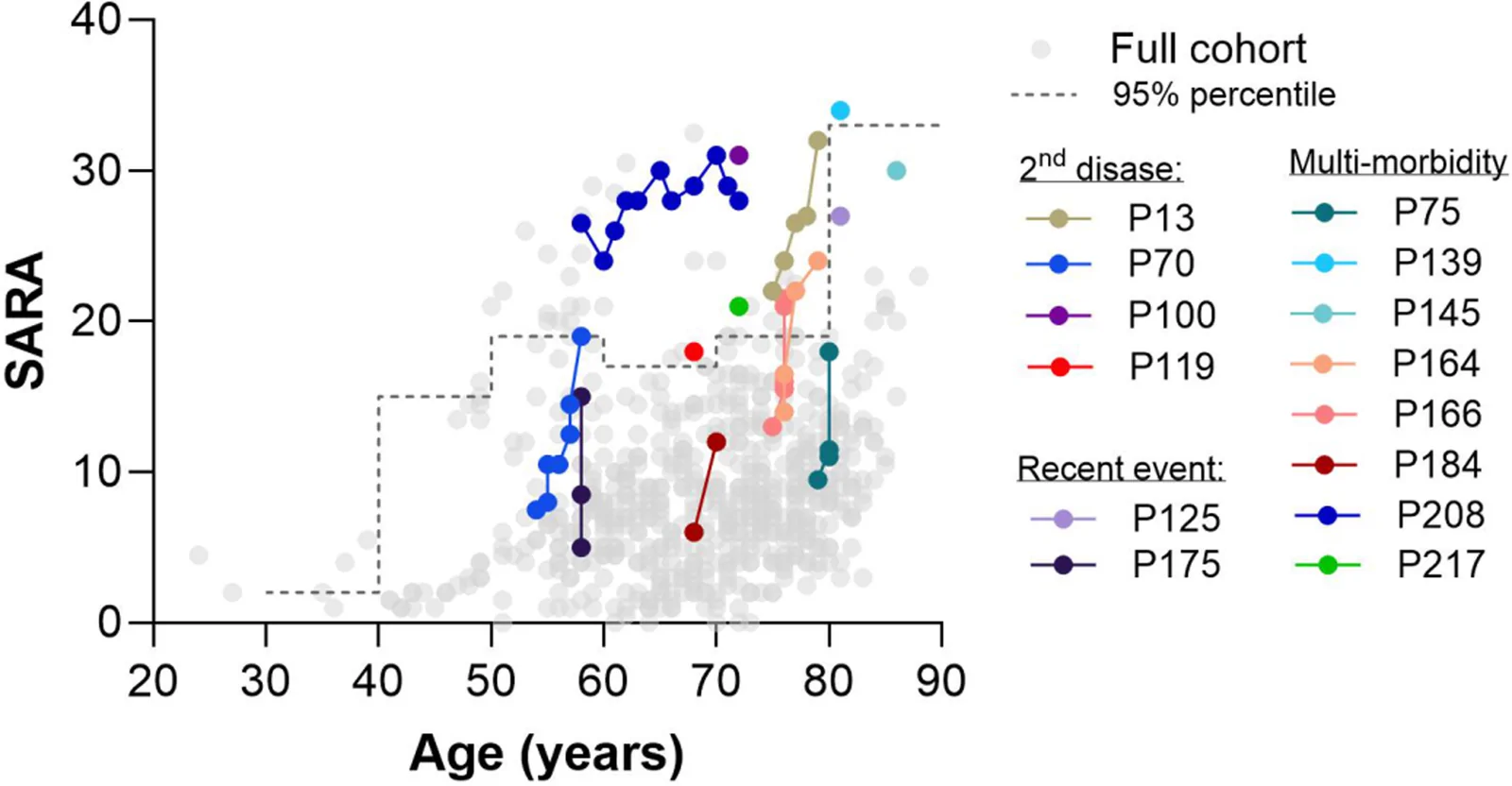
Outlier detection of excess ataxia severity and/or progression in patients with SCA27B and comorbidity affecting motor performance as captured by the SARA. Grey dots reflect scatter of all 661 SARA assessments across 219 patients; dashed line reflects 90th percentile of their distribution calculated per decade of age.

**Fig. 5:**
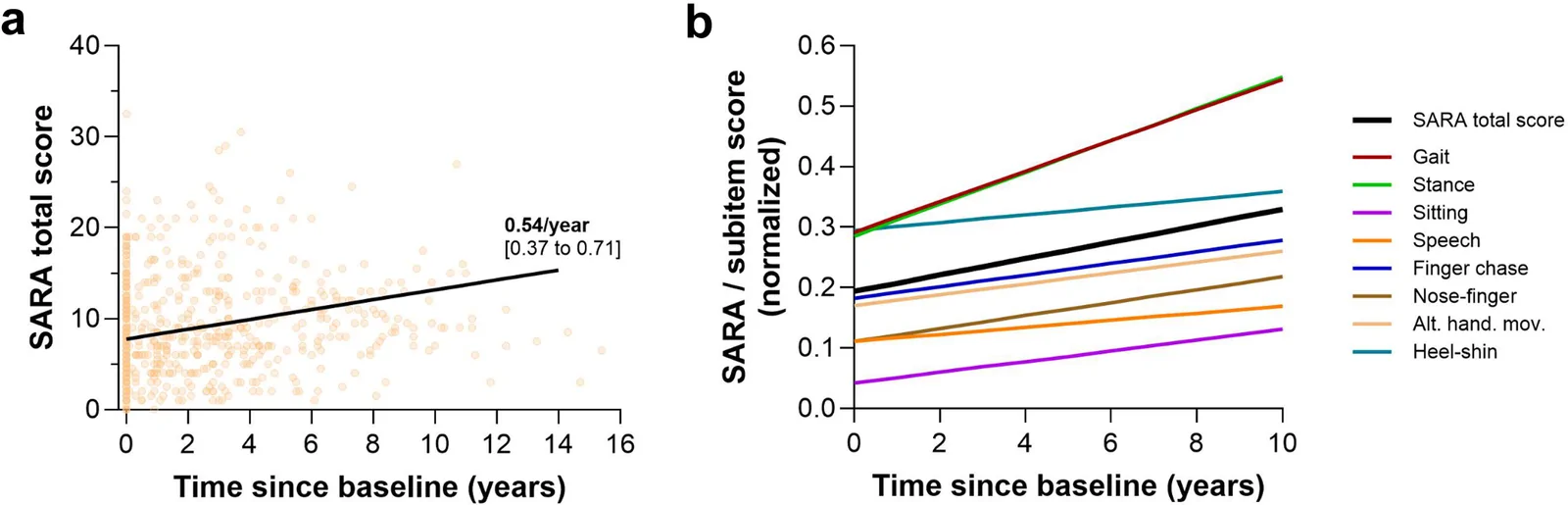
Longitudinal disease progression in SCA27B, as captured by the SARA total score (a) and across ataxia domains by the respective SARA items (b). Lines indicate progression estimate in linear mixed-effect model. Numbers in brackets reflect 95% confidence interval of estimate. Dots in (a) reflect scatter of all 661 SARA assessments across 219 patients with SCA27B, relative to the time since their baseline assessment. Item scores in (b) were normalised to the respective scoring range to allow comparison of progression rates across ataxia domains independent of metric differences of the scale.

Visual inspection of SARA scores across age groups and preliminary progression modelling highlighted several patients with SCA27B with unusually high ataxia severity and/or fast progression (Fig. 4). An in-depth clinical review of 29 patients fulfilling objective outlier criteria (see Methods) revealed significant comorbidities that interfered with the SARA assessment in 14 of these patients (6% of the total SCA27B cohort). These comorbidities included acquired and genetic second diseases (e.g. amyotrophic lateral sclerosis or *RFC1-*associated ataxia), multi-morbidity (e.g. dementia or cancer with reduced functional status), and sequelae of recent events (e.g. falls or surgery; see Table S3 for list of comorbidities). After removing these outliers, the estimated annual progression in SCA27B was 0.54 SARA points/year [95% CI: 0.37–0.71] in the overall cohort (LMEM, *p* < 0.001; Fig. 5A; see Table S4 for details of the model). In the most trial-relevant subcohort of patients still able to walk independently (SARA gait item ≤3), the estimated annual progression was 0.40 SARA points/year [95% CI: 0.26–0.55; *p* < 0.001]. At the item level, two thirds of the overall SARA progression was captured by its items *gait* (0.20 points/year [95% CI: 0.16–0.24], *p* < 0.001) and *stance* (0.16 points/year [95% CI: 0.13–0.19], *p* < 0.001). The relative progression in these items—normalised to their scoring range—exceeded all other items and the total score of the SARA (Fig. 5B; see Table S5 for item-level statistical results), highlighting gait and balance as key ataxia domains affected by longitudinal progression in SCA27B. Sex, age of onset, disease duration, and ataxia severity (SARA score) at baseline were not associated with progression of the total SARA or any of its items (LMEM, all *p* > 0.05; Tables S4 and S5). However, age of onset (i.e. patient age) was associated with higher subscores in items *stance* (0.08 points per decade, [95% CI: 0.01–0.15], *p* = 0.034) and *finger–chase* (0.06 points per decade, [95% CI: 0.02–0.11], *p* = 0.009), thus indicating that stance assessment in SCA27B is susceptible to ageing-related motor impairment independent of its large difference between patients with SCA27B and controls.

### Modification of progression by repeat size of the shorter allele

**Fig. 6:**
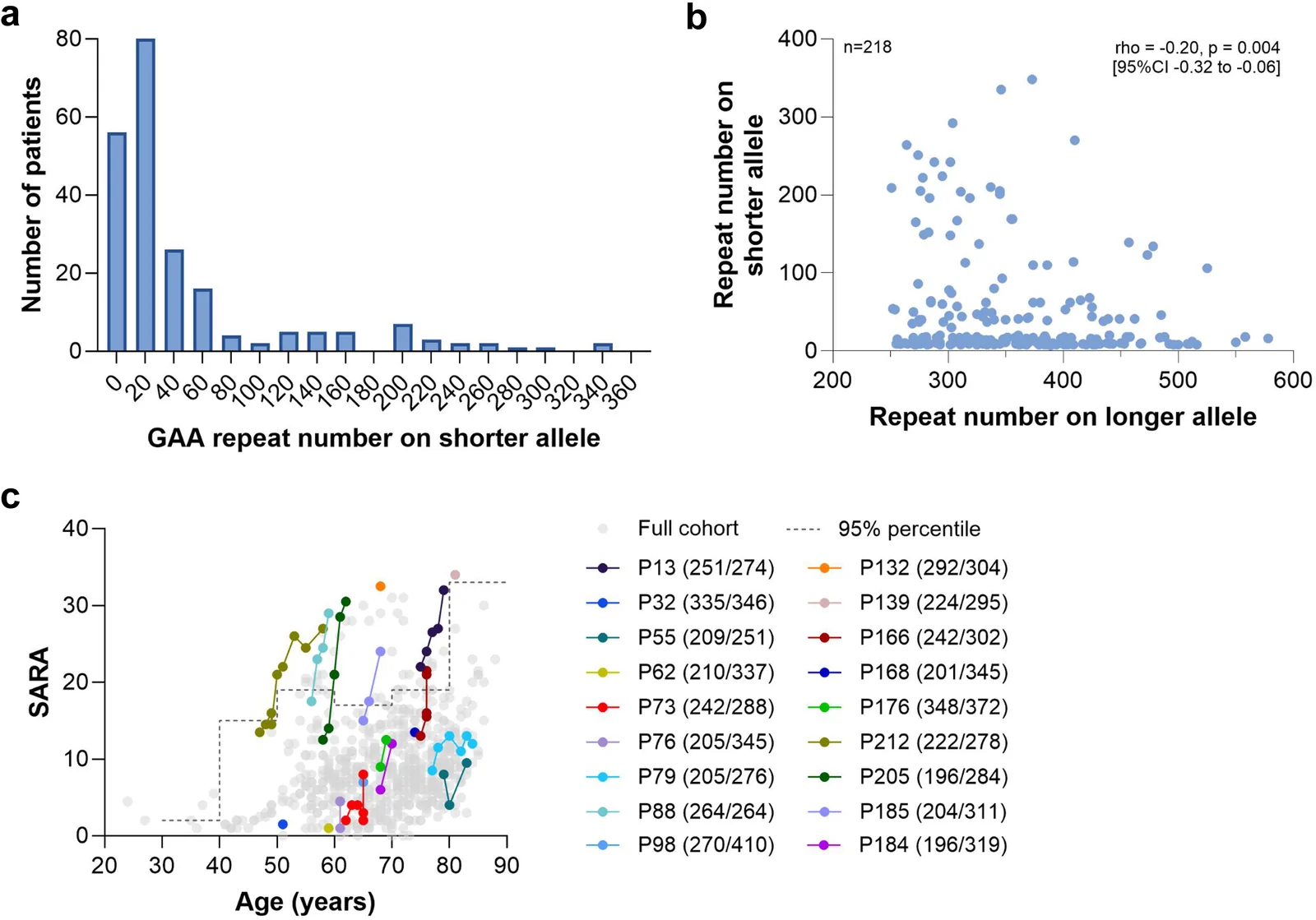
Repeat expansions on the shorter allele of 219 patients with SCA27B. (a) Distribution of repeat numbers on the shorter allele. (b) Spearman correlation of repeat numbers on shorter allele vs. longer allele, excluding one outlier with 937/10 repeats on the longer/shorter allele. (c) Ataxia severity and/or progression in patients with SCA27B and >180 repeat expansions on the shorter allele. Numbers in brackets indicate repeat number on both alleles. Grey dots reflect scatter of all 661 SARA assessments across 219 patients; dashed line reflects 90th percentile of their distribution calculated per decade of age.

Six patients of the cohort showed expanded alleles above the established pathogenic threshold of (GAA)_≥ 250_ repeats also on the shorter allele (range: 251–348), thus carrying bi-allelic pathogenic expansions in *FGF14* (Fig. 6A). Additional twelve patients carried repeat expansions of at least “intermediate size” in the range of (GAA)_180–250_ repeats on the shorter allele (range: 196–242), i.e. above a lower pathogenic threshold of (GAA)_> 180_ repeats recently proposed based on their enrichment in ataxia cohorts.^19^ This yielded a total of 18 patients (8%) in our cohort with (GAA)_> 180_ repeats on the shorter allele, including two patients with also substantial comorbidities (P13: 251/275 repeats; P166: 242/203 repeats). Repeat numbers on the shorter allele were negatively correlated to repeat numbers on the longer allele (Spearman correlation, rho: −0.20, [95% CI: −0.32 to −0.06], *p* = 0.004; n = 218, i.e. excluding one outlier with 937/10 repeats on the longer/shorter allele), i.e. repeat expansions on the shorter allele were not evenly distributed but enriched in patients with shorter expansions on the longer allele (Fig. 6B). GAA repeat number on the shorter allele was not associated with the age of onset (Spearman correlation, rho = 0.004 [95% CI: −0.13–0.14]. *p* = 0.956). However, compared to the full cohort, ataxia severity and/or progression as measured by the SARA appeared higher in patients with bi-allelic (GAA)_≥ 250_ expansions (e.g. P88 or P132), and even also already in patients with (GAA)_> 180_ expansions on the shorter allele (e.g. P205 or P184; Fig. 6C). In LMEM analysis (excluding patients P13 and P16 with comorbidity as possible alternative cause), GAA repeat number on the shorter allele was associated with faster longitudinal disease progression in SCA27B (+0.48 SARA points/year per 100 repeats, [95% CI: 0.19–0.77], *p* = 0.001). Dichotomised by a threshold of (GAA)_> 180_ repeats,^19^ progression in SCA27B was +1.6 SARA points/year faster [95% CI: 0.9–2.2] in patients with GAA repeat numbers on the shorter allele above than below this threshold (LMEM, *p* < 0.001). For the established cutoff of (GAA)_≥ 250_ repeats,^1^^,^^2^^,^^15^ progression was +3.2 points/year faster [95% CI: 1.4–5.0] above than below the threshold (*p* < 0.001). At the SARA item level, the short repeat number was associated with accelerated progression of the most responsive items *gait* and *stance* (by 78% and 47% per 100 repeats relative to progression in the overall cohort, LMEM, *p* < 0.001 and *p* = 0.003); and, in addition, particularly also of the otherwise less responsive items *sitting* or *speech* (by 147% and 174% per 100 repeats; *p* = 0.002 *p* = 0.017; see Table S5 for details).

## Discussion

Leveraging a large cross-sectional and longitudinal multicentre cohort of patients with SCA27B, this study facilitates a deeper understanding of the overall and domain-specific longitudinal disease progression of SCA27B, the metrics of its capture by the SARA, and clinical and in particular genetic modifiers thereof. These results will facilitate a deeper understanding of disease evaluation, and in particular trial planning in this novel, frequent and likely treatable genetic ataxia.

Ataxia severity in SCA27B, as assessed by the SARA, was primarily attributable to impairment in *gait*, *stance*, and lower limb coordination *(heel-shin)*. In contrast, impairment in other ataxia domains contributed substantially less to overall ataxia severity, yielding substantial floor effects and mild subscores ≤1 in SARA score in at least 80% of patients. The strong contribution of gait, stance and lower-limb impairment to overall ataxia severity in SCA27B likely reflects the actual disease phenotype of SCA27B, although capture of gait and balance impairment may be facilitated by the larger number of score levels in items gait and stance than in other SARA items. This might be due to the fact that SCA27B presents as relatively mild ataxia, even across this large cohort of 219 patients (with SARA scores ≤10 in 70% of patients), i.e. in an ataxia disease stage in which genetic ataxias are typically dominated by gait and balance impairment.^22^ It might also point to a major contribution of vestibulo-cerebellar involvement in gait and balance, as suggested earlier.^11^^,^^23^ In contrast, cerebellar networks underlying speech and upper limb control seem to be relatively spared over a long time of the neurodegenerative disease course in most patients with SCA27B, as indicated by a majority of patients with ≤1 point in other SARA items. Correspondingly, to maximise COA sensitivity, our study indicates that future trials in SCA27B should include COAs with higher granularity in the gait and balance domain, e.g. digital gait analysis,^10^ the Upright Stability Scale of the Friedreich Ataxia Rating Scale (FARS-E),^24^ or COAs which focus even stronger on capturing dynamic balance.^25^ These COAs must now be validated in preparation for clinical trials in SCA27B and are thus part of emerging large international multimodal SCA27B natural history studies (ClinicalTrials.gov: NCT06472557).

Our comparison of SARA score distributions between patients with SCA27B and a large cohort of age-stratified controls demonstrated that the effective discrimination between the disease vs. healthy state decreases with age due to ageing-related motor impairment. This is a major caveat for trial planning in SCA27B, given that it is inherently one of the ataxias with the latest, elderly age of onset (average onset in our cohort: 59 years; average onset in published cohorts: 54–68 years^1^, ^2^, ^3^, ^4^, ^5^^,^^8^). Our analysis does not directly infer treatment effect sizes, as the relevant comparator for estimating treatment effects in interventional trials is the counterfactual disease trajectory in untreated patients with SCA27B, rather than the difference to healthy controls. However, the decreasing difference to healthy age-matched controls delineates the maximal SARA delta that a treatment could realistically achieve, as therapeutic interventions are not expected to improve patients beyond the functional level typically observed in healthy individuals of the same age. In a trial scenario, this poorer discrimination would directly correspond to a smaller metric response window of the SARA for a symptomatic treatment effect, e.g. as presumed for 4-AP.^8^^,^^15^

Our item-level comparison showed that this metric interference by ageing-related motor impairment is particularly conveyed by the appendicular items of the SARA, in which ageing-related variability may be indistinguishable (or even exceed) the mild disease-related motor impairment in this domain in SCA27B. This is important in particular for lower-limb coordination, for which the heel-shin item appears to drive SCA27B-related impairment as measured by the SARA, but which becomes susceptible to ageing-related impairment above age 60 years. However, our item-level LMEM demonstrated that age is also associated with poorer performance of stance. Thus, while gait- and balance-centric COAs that omit appendicular ataxia (like the FARS-E^26^ or the fSARA^27^) might probably be overall more responsive than the SARA in SCA27B, they may still be susceptible to age effects in the elderly population suffering from SCA27B. Digital gait analysis may provide better sensitivity in SCA27B if digital measures were specific for ataxia as opposed to healthy ageing, e.g. by capturing gait variability due to cerebellar ataxia as opposed to gait slowing also observed with age,^28^^,^^29^ or dynamic gait aspects like turning^30^ that might be particularly impaired in SCA27B. This highlights the lack of, and at the same time urgent need of, comparative data in healthy aged controls for most clinician-reported and digital outcomes in the ataxia field. Their validation in healthy elderly cohorts will be key for trial planning in SCA27B or similar late-onset ataxias (like e.g. RFC1-disease^31^).

By linear mixed-effect modelling of a large multicentre longitudinal dataset, the present study provides the robust estimate of disease progression in SCA27B. The average progression of 0.54 SARA points/year tends to be higher than previous estimates in smaller, cross-sectional cohorts,^4^^,^^7^^,^^8^ but still falls into a rather mild overall progression rate, lower than most other repeat SCAs (even lower than 0.8 points/year estimated for SCA6^14^) or other genetic ataxias (e.g. 0.6–1.6 points/year for most autosomal recessive cerebellar ataxias).^22^^,^^32^ In contrast to Friedreich Ataxia as another GAA-driven ataxia,^33^ baseline ataxia severity was not a predictor of longitudinal disease progression in SCA27B. Two thirds of this longitudinal progression were contributed by progressive impairment in the gait and stance domain, although the corresponding SARA items gait and stance make up only 35% of the SARA total score. While natural history progression does not directly predict the magnitude or time course of treatment effects, the distinct progression in these domains provides important information on their dynamic range and responsiveness. Domains that show little change over time in untreated disease appear likely to have limited sensitivity to detect change, particularly in disease-modifying trial settings. Our data suggest that gait, stance, and lower-limb components contribute most strongly at least to longitudinal natural history change in SCA27B, thus corroborating and extending their importance from SCA27B ataxia severity (demonstrated by our distribution-based SARA metrics analyses) to their contribution to SCA27B ataxia progression.

Given the above mentioned mild overall disease progression—and the poor sensitivity of clinician-reported outcomes (ClinROs) for ataxia in general,^14^^,^^34^^,^^35^ proving efficacy of disease-modifying treatments will pose an even greater challenge in SCA27B than in other genetic ataxias.^36^ This challenge could even be larger for the most trial relevant target group of patients still able to walk independently (SARA gait item ≤3), in whom estimated progression was only 0.40 SARA points/year. A SARA gait score ≤3 was chosen as a proxy for free, i.e. unassisted ambulation, which represents a trial inclusion criterion in most current ataxia treatment trials testing disease-modifying treatments in SCAs (ClinicalTrials.gov: e.g. NCT05160558, NCT06672445, or NCT02960893). The mild diseases progression emphasises the need for future treatment trials to include: ClinROs more focused on the specific dysfunctions in SCA27B; digital-motor outcomes with demonstrated higher sensitivity to change to gait and balance dysfunction than the SARA (e.g. turning parameters^30^); and/or composite measures combining optimised ClinROs plus optimised digital-motor outcomes.^37^

Our results furthermore indicate that trial inclusion and treatment response capture in SCA27B might be in particular confounded by an inherently high rate of comorbidities in SCA27B. Fourteen of 29 patients, which had an outlier severity and/or progression rate beyond the 95th percentile in the overall cohort, presented with comorbidities as relevant clinical modifiers of disease severity and progression in SCA27B. Exclusion of these patients aimed at minimising the influence of confounding factors that may transiently or permanently distort SARA-based assessments of ataxia severity; and, at the same time, at ensuring robust attribution of observed progression estimates to the underlying disease per se, in particular in heterogeneous real-world settings such as the one analysed here. The high rate of comorbidities seems already a priori reasonable in late-adult populations like SCA27B, but still largely new to the ataxia trial space where the large majority of other SCAs present with a much younger age, and are thus much less confronted with the necessity to exclude patients from trials based on comorbidity-related functional impairment.^38^^,^^39^ Such comorbidities may (i) mask the actual disease severity and/or progression by exceeding the true disease-related motor impairment (e.g. due to independent dementia and small vessel disease [subject P208], neuromuscular disease [P70, P119], or Parkinsonism [P145]); and/or (ii) interfere with trial outcome assessment by unexpected singular events (e.g. hospitalisation or surgery, eventually leading to dropouts). Preparation of trials in SCA27B will thus require rigorous exclusion criteria and in-depth screening for the presence of comorbidities to increase statistical power while maintaining external validity for elderly populations.

Finally, our study also supports GAA repeat expansions also on the *cis shorter* allele, i.e. non-dominant *FGF14* allele, as genetic modifier of SCA27B disease progression. These repeat alleles were estimated to increase longitudinal progression by +0.48 SARA points/year per 100 repeats. This association reflects a relationship in the natural disease trajectory but does not imply it would moderate or predict treatment effects. Rather, it strongly suggests that the short allele must be considered for stratification, adjustment, or enrichment strategies in upcoming trials in SCA27B, similar to what has now become state of the art for Friedreich Ataxia, - interestingly also a GAA repeat expansion (yet autosomal-recessive) disease.^13^^,^^26^ As in Friedreich Ataxia,^40^ larger repeat sizes may be associated with greater transcriptional impairment also in SCA27B, which may contribute to faster disease progression—in an additive manner with the larger allele. At the domain level, expansions on the shorter allele were associated with faster progression in those ataxia domains that dominated ataxia severity in the overall cohort (gait, stance, and lower-limb involvement). Yet, they were particularly associated with greater annual progression also in the domains of sitting and speech by 147% and 174% per 100 repeats, respectively. Based on the distribution of repeat numbers on the shorter allele, 8% of patients with SCA27B carried a second repeat expansion of (GAA)_> 180_ repeats. This lower threshold, here independently suggested by the distribution of repeat numbers on the shorter allele, falls into the range of a lower threshold of (GAA)_> 180_ repeats recently suggested for the longer allele.^19^ The presence of such a shorter allele was associated with an estimated +1.56 SARA points/year faster progression as compared to patients with shorter alleles of (GAA)_< 180_ repeats, thus adding to the pathogenicity of the longer allele. For patients with bi-allelic repeats above the well-established pathogenic cutoff of (GAA) _≥ 250_ repeats, our study estimated even faster progression (+3.2 points/year). This might indicate gradually increasing and synergistic pathogenicity of both alleles in the range of (GAA)_180–250_ repeats. Additional studies are required to characterise the frequency and interaction of bi-allelic repeats in this “intermediate” range, as patients with SCA27B and longer alleles below 250 repeats were not included in our study, and the frequency of bi-allelic repeat expansion carriers is thus probably even higher than our estimate.

Limitations of our study include the retrospective aggregation of SARA assessments; while each of them had been per se prospectively assessed (i.e. not post hoc reconstructed), they had not been prospectively aggregated upfront in a centralised registry. Accordingly, prior rater training was also not a formal requirement in this study, although SARA assessment had been performed predominantly by experienced clinical raters at ataxia expert centres with standardised assessment as part of recruitment into international ataxia registries. Moreover, the SARA does not assess downbeat nystagmus, which is a relevant impairment in SCA27B. Further validation is thus warranted, which is now ongoing in another recently initiated large multimodal international SCA27B natural history study (NCT06472557). While our study includes a large SCA27B cohort, the number of datasets were still too limited to allow for validation of our LMEM by influence in residual diagnostic or by variable selection. The model was thus fitted based on clinical hypotheses based on SCA27B literature and expert experience about likely relevant exploratory factors. Potential attrition bias, assuming a missing-at-random mechanism, was reduced by the LMEM estimation approach. Potential distortion of SARA-based ataxia severity estimates was minimised by screening the available data for outliers in disease severity and/or progression and reviewing the respective clinical records by local SCA27B neurologists for confirmation of the data and/or identification of interfering factors. Future studies in even larger SCA27B cohorts are warranted to confirm our findings, including the frequency of bi-allelic repeat expansion carriers, and in particular to confirm and further delineate clinical and mechanistic effects of shorter alleles >180–200 repeats on SCA27B disease phenotype and progression.

In conclusion, by compiling and analysing a large international cohort of patients with SCA27B, this study provides insights into disease progression trajectories, SARA performance metrics, and genetic modifiers. These findings add to the clinical understanding of SCA27B and offer a basis to inform the design of now incipient treatment trials in this likely treatable ataxia.

## Contributors

AT, RDH, and MS contributed to the conception and design of the study.

AT, FE, CDep, TW, CDel, AN, CA, DP, EI, FH, MR, MB, MGE, JF, TK, LS, BB, MA, DT, and MS contributed to the acquisition of data.

AT, RDH, and MS contributed to the analysis or interpretation of the data.

AT and MS contributed to draughting the text and preparing the figures.

All authors critically reviewed the manuscript for important intellectual content:

AT and MS have directly accessed and verified the underlying data reported in the manuscript. All authors confirm that they had full access to the data in the study and accept responsibility to submit for publication.

All authors read and approved the final version of the manuscript.

## Data sharing statement

Anonymised data underlying this article will be made available upon reasonable request by qualified investigators.

## Declaration of interests

AT, RDH, FE, CDep, TW, CDel, AN, CA, DP, EI, FH, MR, and BB have nothing to disclose.

MB received honoraria from Bial Germany, unrelated to the present manuscript.

MGE received consulting fees from Biogen Germany, and travel support from Aparito, a wholly owned subsidiary of Eli Lilly and Company, all unrelated to this project.

JF has received consultancy honoraria from Vico therapeutics and Biogen, unrelated to the present manuscript.

TK is receiving research support from the Bundesministerium für Forschung, Technologie und Raumfahrt (BMFTR), Servier, and UCB, all unrelated to this project. Within the last 24 months, he has received consulting fees from BottNeuro, Arrowhead, Bristol-Myers Squibb, and UCB, all unrelated to this project.

LS served as consultant for Alexion and Reata Pharmaceuticals unrelated to the present manuscript.

MA received honoraria from IPSEN, Abbvie, Orkyn, Teva, Reata Pharmaceuticals, Biogen, Aguettant, Merz, Ever Pharma, Asdia, all unrelated to the present manuscript.

DT received funding from the DFG, EU and Bernd Fink Foundation, all unrelated to this project.

MS has received consultancy honoraria from Ionis, UCB, Prevail, Orphazyme, Biogen, Servier, Reata, GenOrph, AviadoBio, Biohaven, Zevra, Lilly, Quince, and Solaxa, all unrelated to the present manuscript.

## References

[1] Pellerin D., Danzi M.C., Wilke C. (2023). Deep intronic FGF14 GAA repeat expansion in late-onset cerebellar ataxia. N Engl J Med.

[2] Rafehi H., Read J., Szmulewicz D.J. (2023). An intronic GAA repeat expansion in FGF14 causes the autosomal-dominant adult-onset ataxia SCA50/ATX-FGF14. Am J Hum Genet.

[3] Hengel H., Pellerin D., Wilke C. (2023). As frequent as Polyglutamine spinocerebellar ataxias: SCA27B in a large German autosomal dominant ataxia cohort. Mov Disord.

[4] Wirth T., Clément G., Delvallée C. (2023). Natural history and phenotypic spectrum of GAA-FGF14 sporadic late-onset cerebellar ataxia (SCA27B). Mov Disord.

[5] Iruzubieta P., Pellerin D., Bergareche A. (2023). Frequency and phenotypic spectrum of spinocerebellar ataxia 27B and other genetic ataxias in a Spanish cohort of late-onset cerebellar ataxia. Eur J Neurol.

[6] Kartanou C., Mitrousias A., Pellerin D. (2024). The FGF14 GAA repeat expansion in Greek patients with late-onset cerebellar ataxia and an overview of the SCA27B phenotype across populations. Clin Genet.

[7] Satolli S., Rossi S., Vegezzi E. (2024). Spinocerebellar ataxia 27B: a frequent and slowly progressive autosomal-dominant cerebellar ataxia-experience from an Italian cohort. J Neurol.

[8] Wilke C., Pellerin D., Mengel D. (2023). GAA-FGF14 ataxia (SCA27B): phenotypic profile, natural history progression and 4-aminopyridine treatment response. Brain.

[9] Ashton C., Indelicato E., Pellerin D. (2023). Spinocerebellar ataxia 27B: episodic symptoms and acetazolamide response in 34 patients. Brain Commun.

[10] Seemann J., Traschutz A., Ilg W., Synofzik M. (2023). 4-Aminopyridine improves real-life gait performance in SCA27B on a single-subject level: a prospective n-of-1 treatment experience. J Neurol.

[11] Pellerin D., Heindl F., Wilke C. (2024). GAA-FGF14 disease: defining its frequency, molecular basis, and 4-aminopyridine response in a large downbeat nystagmus cohort. eBioMedicine.

[12] Schmitz-Hübsch T., du Montcel S.T., Baliko L. (2006). Scale for the assessment and rating of ataxia: development of a new clinical scale. Neurology.

[13] Reetz K., Dogan I., Hilgers R.D. (2021). Progression characteristics of the European Friedreich's Ataxia Consortium for Translational Studies (EFACTS): a 4-year cohort study. Lancet Neurol.

[14] Jacobi H., du Montcel S.T., Bauer P. (2015). Long-term disease progression in spinocerebellar ataxia types 1, 2, 3, and 6: a longitudinal cohort study. Lancet Neurol.

[15] Abou Chaar W., Eranki A.N., Stevens H.A. (2024). Clinical, radiological and pathological features of a large American cohort of spinocerebellar ataxia (SCA27B). Ann Neurol.

[16] Kirchberger I., Meisinger C., Heier M. (2012). Patterns of multimorbidity in the aged population. Results from the KORA-Age study. PloS one.

[17] Verghese J., LeValley A., Hall C.B., Katz M.J., Ambrose A.F., Lipton R.B. (2006). Epidemiology of gait disorders in community-residing older adults. J Am Geriatr Soc.

[18] Bonnet C., Pellerin D., Roth V. (2023). Optimized testing strategy for the diagnosis of GAA-FGF14 ataxia/spinocerebellar ataxia 27B. Sci Rep.

[19] Mohren L., Erdlenbruch F., Leitão E. (2024). Identification and characterisation of pathogenic and non-pathogenic FGF14 repeat expansions. Nat Commun.

[20] Grobe-Einsler M., Schmidt A., Schaprian T., Vogt I.R., Klockgether T. (2023). Scale for the assessment and rating of ataxia: age-dependent performance of healthy adults. Eur J Neurol.

[21] Molenberghs G., Verbeke G. (2000).

[22] Traschütz A., Adarmes-Gomez A.D., Anheim M. (2023). Responsiveness of the scale for the assessment and rating of ataxia and natural history in 884 recessive and early onset ataxia patients. Ann Neurol.

[23] Pellerin D., Wilke C., Traschutz A. (2024). Intronic FGF14 GAA repeat expansions are a common cause of ataxia syndromes with neuropathy and bilateral vestibulopathy. J Neurol Neurosurg Psychiatry.

[24] Rummey C., Corben L.A., Delatycki M.B. (2019). Psychometric properties of the Friedreich Ataxia Rating Scale. Neurol Genet.

[25] Powell L.E., Myers A.M. (1995). The activities-specific balance confidence (ABC) scale. J Gerontol A Biol Sci Med Sci.

[26] Rummey C., Corben L.A., Delatycki M. (2022). Natural history of Friedreich ataxia: heterogeneity of neurologic progression and consequences for clinical trial design. Neurology.

[27] Potashman M., Popoff E., Powell L. (2024). Psychometric validation of the modified functional scale for the assessment and rating of ataxia (f-SARA) in patients with spinocerebellar ataxia. Cerebellum.

[28] Ilg W., Seemann J., Giese M. (2020). Real-life gait assessment in degenerative cerebellar ataxia: toward ecologically valid biomarkers. Neurology.

[29] Czech M.D., Psaltos D., Zhang H. (2020). Age and environment-related differences in gait in healthy adults using wearables. NPJ Digit Med.

[30] Thierfelder A., Seemann J., John N. (2022). Real-life turning movements capture subtle longitudinal and Preataxic changes in cerebellar ataxia. Mov Disord.

[31] Traschütz A., Cortese A., Reich S. (2021). Natural history, phenotypic spectrum, and discriminative features of multisystemic RFC1 disease. Neurology.

[32] Traschütz A., Adarmes-Gomez A.D., Anheim M. (2023). Autosomal recessive cerebellar ataxias in Europe: frequency, onset, and severity in 677 patients. Mov Disord.

[33] Manrique L., Martinez-Dubarbie F., Pelayo-Negro A.L. (2026). Longitudinal analysis shows GAA1 length and baseline clinical status as robust predictors of progression in Friedreich ataxia. J Neurol.

[34] Seemann J., Beyme T., John N. (2025). Capture of longitudinal change in real-life walking in cerebellar Ataxia increases patient relevance and effect size. Mov Disord.

[35] Reetz K., Dogan I., Hilgers R.-D. (2016). Progression characteristics of the European Friedreich's Ataxia Consortium for Translational Studies (EFACTS): a 2 year cohort study. Lancet Neurol.

[36] Klockgether T., Synofzik M., AGI working group on COAs and Registries (2024). Consensus recommendations for clinical outcome assessments and registry development in ataxias: Ataxia Global Initiative (AGI) working group expert guidance. Cerebellum.

[37] Hamdan A., Traschutz A., Beichert L. (2025). Integrated modeling of digital-motor and clinician-reported outcomes using item response theory: towards powerful trials for rare neurological diseases. CPT Pharmacometrics Syst Pharmacol.

[38] Lynch D.R., Chin M.P., Delatycki M.B. (2021). Safety and efficacy of omaveloxolone in Friedreich ataxia (MOXIe study). Ann Neurol.

[39] Coarelli G., Heinzmann A., Ewenczyk C. (2022). Safety and efficacy of riluzole in spinocerebellar ataxia type 2 in France (ATRIL): a multicentre, randomised, double-blind, placebo-controlled trial. Lancet Neurol.

[40] Bidichandani S.I., Ashizawa T., Patel P.I. (1998). The GAA triplet-repeat expansion in Friedreich ataxia interferes with transcription and may be associated with an unusual DNA structure. Am J Hum Genet.

