# Supplementary material for: Longitudinal progression, metrics, age-dependence, and modifiers of ataxia severity in SCA27B: a multicentre study of 219 patients
Source: eBioMedicine. 2026 Aug 15;131:106437. doi: 10.1016/j.ebiom.2026.106437 (PMC13503081; doi:10.1016/j.ebiom.2026.106437)
Supplement: Supplementary file 1 [file mmc1.docx]

**Supplementary Material**

**Table S1: Distribution of SCA27B cohort characteristics and prevalence of non-ataxia features at baseline visit across sites**

|  | **Total Cohort** | Berlin, Germany | Essen, Germany | Innsbruck, Austria | Lübeck, Germany | Montreal,  Canada | Munich, Germany | Nancy, France | Strasbourg,  France | Tübingen, Germany |
| --- | --- | --- | --- | --- | --- | --- | --- | --- | --- | --- |
| Patients screened with SCA27B | 366 | 23 | 34 | 10 | 14 | 107 | 83 | 15 | 26 | 54 |
| Patients included with item-level SARA | **219** | **21** | **34** | **10** | **7** | **44** | **12** | **11** | **26** | **54** |
| Age (years) | 68±10  (24-86) | 67±11  (41-80) | 61±11  (24-81) | 68±7  (56-79) | 69±9 (52-77) | 68±10  (43-86) | 67±10  (43-79) | 72±8  (59-85) | 74±6  (58-85) | 68±10  (36-83) |
| Females | 101 (46%) | 10 (48%) | 19 (56%) | 3 (30%) | 2 (29%) | 21 (48%) | 7 (58%) | 7 (64%) | 8 (31%) | 24 (44%) |
| Age of onset (years) | 59±12  (21-79) | 58±12  (30-76) | 52±14  (21-76) | 59±8  (50-70) | 64±10  (46-75) | 55±11  (36-79) | 60±13  (33-77) | 64±9  (50-76) | 69±7  (52-79) | 59±12  (28-78) |
| Disease duration (years) | 9±7  (0-32) | 8±7  (1-25) | 9±7  (2-28) | 9±5  (2-16) | 6±4  (2-14) | 13±8  (1-32) | 7±5  (2-17) | 9±6  (2-21) | 5±4  (1-15) | 10±7  (0-26) |
| Ataxia severity (SARA score) | 9±6  (0-34) | 6±3  (2-12) | 7±6  (0-27) | 12±6  (5-24) | 8±7  (0-21) | 10±9  (0-34) | 4±4  (1-13) | 12±9  (2-31) | 10±5  (4-22) | 9±5  (1-22) |
| Number of FGF14 GAA repeats |  |  |  |  |  |  |  |  |  |  |
| Longer allele | 356  [303-407] | 332  [277-422] | 326  [280-411] | 377  [345-428] | 340  [275-440] | 378  [327-424] | 343  [273-426] | 410  [290-510] | 349  [303-385] | 346  [311-403] |
| Shorter allele | 16  [9-53] | 17  [10-129] | 18  [10-49] | 47  [16-169] | 40  [10-50] | 11  [9-34] | 13  [8-121] | 11  [9-37] | 34  [11-79] | 16  [8-55] |
| Drug treatment |  |  |  |  |  |  |  |  |  |  |
| On 4-AP or 3,4-DAP | 40 (18%) | 8 (38%) | 11 (32%) | 1 (10%) | 0 (0%) | 1 (2%) | 7 (58%) | 0 (0%) | 0 (0%) | 12 (22%) |
| On Acetazolamide | 14 (6%) | 2 (10%) | 2 (6%) | 6 (60%) | 0 (0%) | 2 (5%) | 0 (0%) | 1 (9%) | 0 (0%) | 1 (2%) |
| Longitudinal follow-up |  |  |  |  |  |  |  |  |  |  |
| Number of patients | 116 (53%) | 21 (100%) | 26 (77%) | 6 (60%) | 1 (14%) | 7 (16%) | 5 (42%) | 1 (9%) | 24 (92%) | 25 (46%) |
| Number of follow-up visits | 4 [3-6] | 3 [5-7] | 5 [3-8] | 4 [3-4] | 4 | 3 [2-3] | 3 [2-5] | 2 | 5 [3-7] | 3 [2-5] |
| Follow-up time (years) | 4·7±3·4  (0·3-15·4) | 4·5±3·6 (0·4-12·3) | 7·1±4·0  (1·5-15·4) | 4·0±1·5 (3·0-7·0) | 2·7 | 4·5±1·6 (1·9-6·3) | 1·2±1·5 (0·3-3·8) | 1.1 | 4·4±3·3 (0·4-10·0) | 4·3±2·8 (0·9-10·2) |
|  | **Total Cohort** | Berlin, Germany | Essen, Germany | Innsbruck, Austria | Lübeck, Germany | Montreal,  Canada | Munich, Germany | Nancy, France | Strasbourg,  France | Tübingen, Germany |
| Non-ataxia features (per assessed patient) |  |  |  |  |  |  |  |  |  |  |
| Impaired vibration at ankle  (predominantly mild to moderate) | 93/215 (43%) | 5/21 (24%) | 9/33 (27%) | 5/10 (50%) | 6/7 (86%) | 15/43 (35%) | 5/11 (46%) | 9/11 (82%) | 12/25 (48%) | 27/54 (50%) |
| Impaired head impulse test | 20/100 (20%) | 5/21 (24%) | 1/8 (13%) | 2/10 (20%) | 6/7 (86%) | 1/1 (100%) | 2/11 (18%) | not assessed | 0/26 (0%) | 3/16 (19%) |
| Pyramidal signs (hyperreflexia or   spasticity in the legs or extensor plantar) | 16/216 (7%) | 0/20 (0%) | 1/34 (3%) | 3/10 (30%) | 0/7 (0%) | 3/43 (7%) | 0/11 (0%) | 3/11 (27%) | 3/26 (12%) | 3/54 (6%) |
| Paresis   (unspecific proximal leg weakness) | 3/218 (1%) | 0/21 (0%) | 1/34 (3%) | 0/10 (0%) | 0/7 (0%) | 1/44 (2%) | 0/12 (0%) | 0/10 (0%) | 0/26 (0%) | 1/54 (20%) |
| Hyperkinetic movement disorders  (mild postural or head tremor) | 12/216 (6%) | 3/21 (14%) | 1/34 (3%) | 0/10 (0%) | 0/7 (0%) | 5/41 (12%) | 0/12 (0%) | 0/11 (0%) | 0/26 (0%) | 3/54 (6%) |
| Parkinsonism  (mild bradykinesia ± rigidity) | 11/219 (5%) | 1/21 (5%) | 0/34 (0%) | 0/10 (0%) | 0/7 (0%) | 1/44 (2%) | 1/12 (8%) | 2/11 (18%) | 3/26 (12%) | 3/54 (6%) |
| Cognitive impairment  (predominantly MCI) | 19/216 (8%) | 0/21 (0%) | 5/31 (16%) | 2/10 (20%) | 1/7 (14%) | 2/44 (5%) | 1/12 (8%) | 2/11 (18%) | 3/26 (12%) | 3/54 (6%) |

Data presented as mean ± standard deviation (range), median [interquartile range] or number of patients (%cohort). 4-AP = 4-aminopyridine; 3,4-DAP = 3,4-diaminopyridine.

**Figure S2: Cross-sectional progression as measured by the SARA at the item level.**

Linear regression with slope [95% confidence interval] of cross-sectional progression as measures by the SARA total score.
Rho: Spearman correlation [95% confidence interval]

**Table S3: SCA27B patients with comorbidity excluded for longitudinal progression modelling**

| **Patient identifier** | **Outlier criterion** | **Exclusion criterion  (after manual review of clinical records)** |
| --- | --- | --- |
| 13 | >95% SARA by age | Probable 2^nd^ disease due to atypical phenotype with cognitive impairment, severe bradykinesia, spastic paraparesis, and global atrophy on MRI |
| 70 | >95% slope in LMEM | Confirmed 2^nd^ disease: Amyotrophic lateral sclerosis |
| 75 | >95% slope in LMEM | Multi-morbidity: cervical compressive myelopathy and complicated hip replacement with infection during the assessment period |
| 100 | >95% SARA in LMEM | Confirmed 2^nd^ disease: CANVAS due to bi-allelic RFC1 repeat expansions |
| 119 | >95% SARA by age | Confirmed 2^nd^ disease: Lambert-Eaton myasthenic syndrome |
| 125 | >95% SARA in LMEM | Recent event: follow-up assessment after hospitalization and deconditioning with severe loss of mobility after fall |
| 139 | >95% SARA in LMEM | Multi-morbidity: advanced mixed dementia (Alzheimer’s disease + vascular) with severe bilateral visual loss due to prior intracranial hemorrhage |
| 145 | >95% SARA in LMEM | Multi-morbidity: history of breast cancer with chemotherapy, severe diabetes, and parkinsonism treated with levodopa |
| 164 | >95% slope in LMEM | Multi-morbidity: depression, acromioplasty, severe peripheral artery occlusive disease, and poor performance status due to chronic myeloid leukemia |
| 166 | >95% slope in LMEM | Multi-morbidity: poor performance status due to adenocarcinoma of the lung, chemotherapy and toxic neuropathy |
| 175 | >95% slope in LMEM | Recent event: total right hip replacement |
| 184 | >95% slope in LMEM | Multi-morbidity and recent event: history of knee and hip replacement, recurrent falls with radial and spinal fractures, and recent fall prior to follow-up assessment |
| 208 | >95% SARA in LMEM | Multi-morbidity: dementia, epilepsy with focal and secondary generalized seizures, infratentorial meningioma, and severe small vessel disease |
| 217 | >95% SARA by age | Multi-morbidity: poor functional status due to alcohol abuse, chronic obstructive pulmonary disease, peripheral arterial occlusive disease, heart and kidney failure |

CANVAS = Cerebellar ataxia, neuropathy, and vestibular areflexia syndrome; LMEM = Linear mixed effect model; SARA = Scale for the Assessment and Rating of Ataxia.

**Table S4: Linear mixed-effect model for estimation of disease progression in SCA27B**

| **Effect** | **Estimate** | **Standard Error** | **DF** | **t Value** | **Pr > \|t\|** | **Alpha** | **Lower 95%CI** | **Upper**  **95%CI** |
| --- | --- | --- | --- | --- | --- | --- | --- | --- |
| **Intercept** | 7·7475 | 0·1403 | 288 | 55·23 | <·0001 | 0·05 | 7·4715 | 8·0236 |
| **SARA at baseline** | 0·9614 | 0·01966 | 299 | 48·91 | <·0001 | 0·05 | 0·9228 | 1·0001 |
| **Time since baseline**  (years) | 0·5412 | 0·08700 | 73·5 | 6·22 | <·0001 | 0·05 | 0·3678 | 0·7145 |
| **Age of onset**  (years) | -0·00179 | 0·009759 | 275 | -0·18 | 0·8646 | 0·05 | -0·02100 | 0·01742 |
| **Disease duration**  (years) | 0·003572 | 0·01881 | 279 | 0·19 | 0·8495 | 0·05 | -0·03346 | 0·04061 |
| **Patient on 4-AP** | -0·4415 | 0·2611 | 485 | -1·69 | 0·0915 | 0·05 | -0·9544 | 0·07148 |
| **Patient off 4-AP** | 0 | · | · | · | · | · | · | · |
| **GAA repeat number on longer allele** | 0·001001 | 0·001226 | 270 | 0·82 | 0·4150 | 0·05 | -0·00141 | 0·003414 |
| **GAA repeat number  on shorter allele** | 0·001600 | 0·001459 | 282 | 1·10 | 0·2739 | 0·05 | -0·00127 | 0·004473 |
| **Sex** (male) | 0·3229 | 0·1886 | 270 | 1·71 | 0·0881 | 0·05 | -0·04850 | 0·6943 |
| **Sex** (female) | 0 | · | · | · | · | · | · | · |
| **Time since baseline  x SARA at baseline** | 0·01440 | 0·01995 | 83·7 | 0·72 | 0·4725 | 0·05 | -0·02527 | 0·05407 |
| **Time since baseline  x Age of onset** | 0·008123 | 0·008418 | 80·4 | 0·97 | 0·3374 | 0·05 | -0·00863 | 0·02487 |
| **Time since baseline  x Disease duration** | -0·00253 | 0·01673 | 76·2 | -0·15 | 0·8803 | 0·05 | -0·03584 | 0·03079 |
| **Time since baseline  x GAA repeat size on  shorter allele** | 0·004802 | 0·001437 | 78·2 | 3·34 | 0·0013 | 0·05 | 0·001941 | 0·007663 |

4-AP = 4-aminopyridine; CI = confidence interval; SARA = Scale for the Assessment and Rating of Ataxia.

**Table S5: Linear mixed-effect modeling of item-level SARA progression in SCA27B**

| **SARA item:** | **Intercept** | **Age of Onset** (per 10 yrs) | **Disease Duration**  (per yr) | **Overall progression** (pts/year) | **Progression x Age of Onset**  (per 10 yrs) | **Progression x Disease Duration** (per yr) | **Progression x Repeat size on short allele**  (per 100 repeats) |
| --- | --- | --- | --- | --- | --- | --- | --- |
| **1: Gait** | 2·33 [2·21 to 2·46] | 0·026  [-0·068 to 0·120]  p = 0·58 | 0·009  [-0·009 to 0·028]  p = 0·31 | 0·202  [0·160 to 0·244]  **p < 0·001** | 0·035  [-0·003 to 0·074]  p = 0·07 | 0·002  [-0·006 to 0·009]  p = 0·67 | 0·158  [0·094 to 0·224]  **p < 0·001** |
| **2: Stance** | 1·71  [1·64 to 1·78] | 0·075  [0·006 to 0·145]  **p = 0·03** | 0·018  [0·004 to 0·031]  **p = 0·01** | 0·158  [0·126 to 0·189]  **p < 0·001** | 0·027  [-0·002 to 0·056]  p = 0·07 | 0·002  [-0·003 to 0·007]  p = 0·43 | 0·074  [0·025 to 0·124]  **p = 0·003** |
| **3: Sitting** | 0·17  [0·11 to 0·23] | 0·019  [-0·026 to 0·065]  p = 0·41 | 0·008  [-0·001 to 0·016]  p = 0·09 | 0·036  [0·015 to 0·057]  **p = 0·001** | 0·010  [-0·010 to 0·029]  p = 0·33 | -0·001  [-0·004 to 0·003]  p = 0·76 | 0·053  [0·020 to 0·086]  **p = 0·002** |
| **4: Speech** | 0·67  [0·57 to 0·76] | 0·027  [-0·041 to 0·095]  p = 0·44 | 0·009  [-0·004 to 0·023]  p = 0·17 | 0·035  [0·004 to 0·066]  **p = 0·03** | 0·020  [-0·010 to 0·049]  p = 0·18 | 0·0001  [-0·005 to 0·006]  p = 0·97 | 0·061  [0·011 to 0·110]  **p = 0·02** |
| **5: Finger-chase** | 0·73  [0·67 to 0·79] | 0·060  [0·015 to 0·106]  **p = 0·01** | 0·012  [0·003 to 0·021]  **p = 0·01** | 0·039  [0·018 to 0·059]  **p < 0·001** | -0·012  [-0·031 to 0·007]  p = 0·22 | -0·001  [-0·004 to 0·003]  p = 0·75 | 0·031  [-0·002 to 0·064]  p = 0·07 |
| **6: Nose-finger** | 0·44  [0·38 to 0·51] | 0·028  [-0·018 to 0·074]  p = 0·23 | 0·010  [0·001 to 0·019]  **p = 0·03** | 0·043  [0·022 to 0·064]  **p < 0·001** | 0·011  [-0·008 to 0·030]  p = 0·26 | 0·0001  [-0·002 to 0·005]  p = 0·52 | 0·026  [-0·008 to 0·059]  p = 0·12 |
| **7: Altern. hand mov.** | 0·68  [0·62 to 0·74] | 0·031  [-0·015 to 0·076]  p = 0·19 | 0·011  [0·002 to 0·020]  **p = 0·02** | 0·036  [0·015 to 0·057]  **p < 0·001** | 0·008  [-0·011 to 0·028]  p = 0·40 | 0·001  [-0·003 to 0·005]  p = 0·58 | 0·008  [-0·024 to 0·041]  p = 0·61 |
| **8: Heel-shin** | 1·18  [1·12 to 1·24] | -0·008  [-0·054 to 0·037]  p = 0·72 | 0·005  [-0·004 to 0·014]  p = 0·24 | 0·026  [0·005 to 0·047]  **p = 0·02** | 0·010  [-0·009 to 0·029]  p = 0·31 | -0·0004  [-0·004 to 0·003]  p = 0·83 | 0·055  [0·022 to 0·088]  **p = 0·001** |

SARA = Scale for the Assessment and Rating of Ataxia.
